# Comparative Genomic Analysis among Four Representative Isolates of *Phytophthora sojae* Reveals Genes under Evolutionary Selection

**DOI:** 10.3389/fmicb.2016.01547

**Published:** 2016-09-30

**Authors:** Wenwu Ye, Yang Wang, Brett M. Tyler, Yuanchao Wang

**Affiliations:** ^1^Department of Plant Pathology, Nanjing Agricultural UniversityNanjing, China; ^2^Center for Genome Research and Biocomputing, and Department of Botany and Plant Pathology, Oregon State University, CorvallisOR, USA

**Keywords:** *Phytophthora*, comparative genomic analysis, positive selection, effectors, evolution

## Abstract

Comparative genomic analysis is useful for identifying genes affected by evolutionary selection and for studying adaptive variation in gene functions. In *Phytophthora sojae*, a model oomycete plant pathogen, the related study is lacking. We compared sequence data among four isolates of *P. sojae*, which represent its four major genotypes. These isolates exhibited >99.688%, >99.864%, and >98.981% sequence identities at genome, gene, and non-gene regions, respectively. One hundred and fifty-three positive selection and 139 negative selection candidate genes were identified. Between the two categories of genes, the positive selection genes were flanked by larger intergenic regions, poorly annotated in function, and less conserved; they had relatively lower transcription levels but many genes had increased transcripts during infection. Genes coding for predicted secreted proteins, particularly effectors, were overrepresented in positive selection. Several RxLR effector genes were identified as positive selection genes, exhibiting much stronger positive selection levels. In addition, candidate genes with presence/absence polymorphism were analyzed. This study provides a landscape of genomic variation among four representative *P. sojae* isolates and characterized several evolutionary selection-affected gene candidates. The results suggest a relatively covert two-speed genome evolution pattern in *P. sojae* and will provide clues for identification of new virulence factors in the oomycete plant pathogens.

## Introduction

Oomycetes form a distinct phylogenetic lineage of fungus-like eukaryotic microorganisms and belong to the kingdom *Stramenopila*, which also includes brown algae and diatoms (Beakes et al., 2012). The *Phytophthora* genus contains more than 100 described species (Kroon et al., 2012), of which many are plant pathogens of considerable economic importance. For example, *P. infestans* and *P. ramorum* are responsible for the late blight of potato (*Solanum tuberosum* L.) and sudden oak death, respectively. The soybean (*Glycine max* L.) root and stem rot agent, *P. sojae*, has also caused considerable problems for the agricultural industry.

Genome sequences of the hemibiotrophic *P. sojae* (Tyler et al., 2006), *P. ramorum* (Tyler et al., 2006), *P. infestans* (Haas et al., 2009), *P. capsici* (Lamour et al., 2012), and *P. litchii* (Ye et al., 2016) have been made available and analyzed. Accompanying the genomes of phytopathogenic species in other oomycete genera, such as the obligate biotrophic *Hyaloperonospora arabidopsidis* (Baxter et al., 2010) and *Albugo* spp. (Kemen et al., 2011; Links et al., 2011), and the necrotrophic *Pythium* spp. (Levesque et al., 2010; Adhikari et al., 2013), the data have revealed striking variation in genome size and content, a plastic set of pathogenesis-associated genes, and adaptation related to trophic modes, thereby contributing to a greatly accelerated study of host–pathogen interactions (Jiang and Tyler, 2012; Judelson, 2012).

Comparing genomes of different organisms is ideal to identify genes affected by evolutionary selection. For example, genes with reduced or increased substitution rates, which might result from negative selection (NS or purifying selection) or positive selection (PS or adaptive selection), respectively, could be identified (Petersen et al., 2007). For example, those genes identified as effector genes provide evidence for adaptive changes in functions. As a focus of research on host–pathogen interactions, effectors are proteins secreted by pathogens that alter the structure and function of host cells. They either facilitate infection (virulence factors and toxins), trigger defense responses (avirulence factors and elicitors), or both (Hogenhout et al., 2009). As a result, effector genes might be major targets of natural selection upon coevolution of host and pathogen (Ma and Guttman, 2008; McCann and Guttman, 2008; Hogenhout et al., 2009; Stukenbrock et al., 2011; Rech et al., 2014). Many effector genes evolve at accelerated rates compared to the core genome of pathogens. These genes often display extreme levels of PS, with significantly higher rates of non-synonymous to synonymous nucleotide substitutions (Ka/Ks or dN/dS ratios greater than 1) (Ma et al., 2006; Win and Kamoun, 2008; Hogenhout et al., 2009; Wang et al., 2011; Dong et al., 2012).

*Phytophthora sojae* is a model oomycete species. The isolates P6497 (race 2), P7064 (race 7), P7074 (race 17), and P7076 (race 19) comprise its four major genotypes, and encompass nearly all of the known genetic variations (Forster et al., 1994). Genomes of the four isolates have been sequenced. P6497 was the first genome-sequenced *P. sojae* isolate and also the first case among oomycete plant pathogens (Tyler et al., 2006); genome assemblies of P7064, P7074, and P7076 have also become available (Wang et al., 2011). Based on these data, high levels of polymorphisms and significant evidence for PS have been identified from many RxLR (Wang et al., 2011), NLP (Dong et al., 2012), and CRN (Shen et al., 2013) effector genes. However, the type and number of additional genes affected by evolutionary selection in the whole genome are unknown, and a comprehensive genome comparison among the four representative isolates of *P. sojae* is lacking.

In this study, we compared genome sequences of the four representative *P. sojae* isolates and revealed the degree of sequence variation at the level of the whole genome. Candidate genes under PS and NS were characterized and compared for annotated functions and transcription patterns.

## Materials and Methods

### Sequence and Associated Analysis

The *P. sojae* P6497 sequences (v1.1) were obtained from the DOE Joint Genome Institute (JGI) database^1^. The *P. sojae* P7064, P7074, and P7076 sequences were obtained from eumicrobedb.org (formerly VBI Microbial Database). The sequence mapping among genome assemblies and identification of allele sequences were based on the BLAST program (Altschul et al., 1990). The detailed analysis is illustrated in **Supplementary Figure S1**.

To construct the ML tree, the sequences of 2,626 allele groups were aligned using MUSCLE (Edgar, 2004). The aligned gene sequences from each isolate were then concatenated in order, into a super-sequence, and PhyML implemented in the SEAVIEW was used to construct the ML tree using default parameters (Gouy et al., 2010).

To identify genes under PS or NS, the allele sequences of four *P. sojae* isolates were aligned using MUSCLE (Edgar, 2004). The stop codons and codons corresponding to gaps in the alignment were removed. The dN and dS for each pair of alleles, between P6497 and one of the other three isolates, were calculated using YN00 from the PAML package (Yang, 2007). Statistical significance of the differences between dN and dS was based on standard errors computed from YN00 for a Student’s *t*-test. The pair of alleles with a *P-*value < 0.1 and dN/dS > 1.2 or < 1/1.2 were designated as preliminary candidate PS and NS genes, respectively. A *P*-value < 0.05 (after Bonferroni correction) and dN/dS > 2 were used as threshold for candidate genes with stronger PS.

### Functional Annotation and Transcription Profiling

Annotated GO terms were downloaded from the JGI database^1^ (*P. sojae* v1.1), and the distribution of genes in different categories of slimed GO terms were compared and plotted using WEGO (Ye et al., 2006). Detailed functions or functional domains of the identified genes were further annotated using NCBI BLASTP (Altschul et al., 1990) and CDD (Marchler-Bauer et al., 2011). The transcription data were collected from the *Phytophthora* transcriptional database (PTD v1.1^2^) (Ye et al., 2011) and analyzed using MEV (Saeed et al., 2003). The secreted proteins were predicted using SignalP 2.0 and 3.0, TargetP 1.1, and TMHMM 2.0 in www.cbs.dtu.dk/services (Nielsen and Krogh, 1998; Emanuelsson et al., 2000; Krogh et al., 2001). The defined secretome should simultaneously have a SignalP HMM score >0.9 (prediction of signal peptide), subcellular localization as secreted (TargetP; default parameters), and no transmembrane domain after signal peptide cleavage sites (TMHMM; default parameters).

## Results

### Sequence Alignment among the Four *P. sojae* Genome Assemblies

As a reference, we used the genome data of the *P. sojae* isolate P6497, which is 95 Mb in assembly size and contains 19,027 predicted genes (Tyler et al., 2006). Genome assemblies of the other three representative *P. sojae* isolates (P7064, P7074, and P7076) have also become available from 454 resequencing. The isolates P7064, P7074, and P7076 are estimated to cover 12.7-, 6.8-, and 13.2-fold of the genome, contain 38,548, 49,012, and 25,362 contigs, and are 64.0, 51.3, and 60.1 Mb in size, respectively (Wang et al., 2011).

**FIGURE 1:**
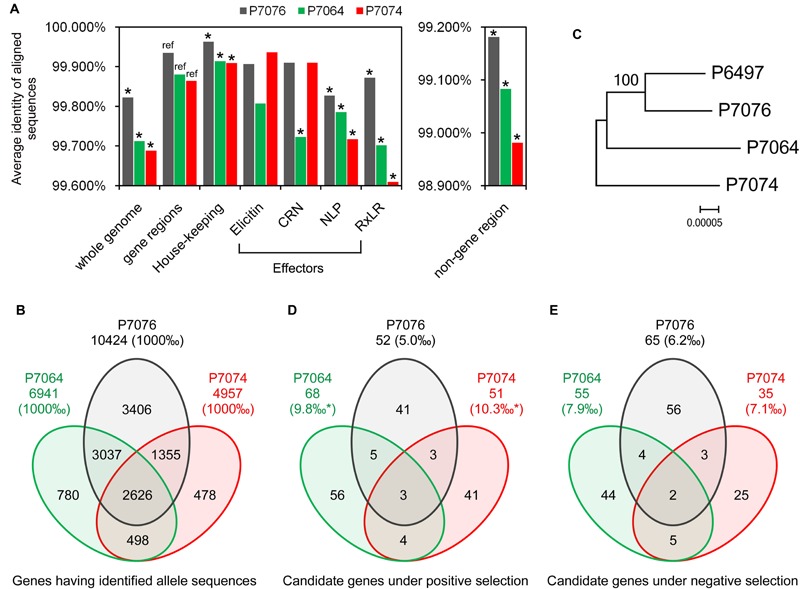
**Identification and polymorphism of the *P. sojae* alleles. (A)** Average sequences identities of the whole genome, gene regions, and non-gene regions, using P6497 as a reference. Asterisk indicates a *P* < 0.001 from a hypergeometric test between the identity values, using value of gene regions as a reference. **(B)** Venn diagram showing the numbers of identified alleles in one or several isolate(s). **(C)** The Maximum-Likelihood phylogenetic tree was constructed based on super sequences that concatenated from 2626 genes, which had identified allele sequences in all of the four isolates. **(D,E)** Venn diagrams showing the numbers of candidate PS and NS genes in one or several isolate(s), respectively. Asterisk indicates a *P* < 0.001 from a χ^2^ test between the proportion values, using value of P7076 as a reference.

Based on the BLASTN program with an *e*-value of 1e-20 as a cut-off, the contig sequences of P7064, P7074, and P7076 were aligned against the scaffold sequences of P6497, and 95.0, 95.9, and 99.5%, respectively, of the contigs could be mapped to the referenced P6497 scaffolds. To increase mapping accuracy, the contigs with multiple best matches (with the same highest BLASTN scores) and/or incomplete alignment coverage (with <98% coverage on the queried contig) were discarded. The remaining 78.8, 81.8, and 78.7% of the contigs were suggested to have high mapping quality for P7064, P7074, and P7076, respectively. The flowchart and statistics of data processing are provided in **Supplementary Figure S1** and Supplementary Table S1, respectively, and the detailed mapping data are in Supplementary Table S2. Average identities of these genome sequences aligned between *P. sojae* isolate pairs were calculated to be 99.688% for P7074 vs. P6497, 99.712% for P7064 vs. P6497, and 99.822% for P7076 vs. P6497 (**Figure 1A**). The genome sequences among the four *P. sojae* isolates were nearly identical, but P7076 was closest to P6497, and P7074 was most distant from P6497 and quite close to P7064.

### Sequence Polymorphism among the *P. sojae* Alleles

To identify the allele sequences of *P. sojae* genes from the P7064, P7074, and P7076 assemblies, genome nucleotide sequences (including introns and containing no ambiguous sequences [i.e., no sequences other than A, T, C, and G]) of 18,568 predicted genes in *P. sojae* P6497 were used as a reference (Tyler et al., 2006). The gene sequences were aligned against the contigs of the other three isolates using BLASTN, with an *e*-value of 1e-20 as a cut-off. Almost all of the genes had matched sequences in the three isolates; there were between 18,475 (99.5%) and 18,526 (99.8%) genes (Supplementary Table S1). However, due to 40% of the 18,568 gene sequences had at least one non-self hit among themselves (based on an all vs. all BLASTN, with an *e*-value of 1e-40 as a cut-off), we reserved candidate allele sequences that were only from the high-quality mapping contigs (the results in Supplementary Table S2) to avoid a bias from false mapping to paralogous genes. In addition, these contigs should at least have complete alignment coverage on the full length of the referenced genes in P6497 (**Supplementary Figure S1**). Finally, we obtained 6,941, 4,957, and 10,424 candidate allele sequences from the P7064, P7074, and P7076 contigs, respectively, and alleles for 2,626 P6497 genes could be identified in the other three isolates (**Figure 1B**, Supplementary Table S3).

When comparing coding DNA sequences (CDSs) between the identified allele pairs, we found 3,467 for P7064 vs. P6497 (49.9% of all identified allele pairs), 2,447 for P7074 vs. P6497 (49.4%), and 3,227 for P7076 vs. P6497 (31.0%) that were polymorphic (Supplementary Table S3). The majority of allele pairs had no more than three mismatches or gaps (**Supplementary Figure S2**). Of all the allele pairs, the average sequence identities were 99.864% for P7074 vs. P6497, 99.880% for P7064 vs. P6497, and 99.935% for P7076 vs. P6497. They were all greater than the values of genome sequence comparisons (**Figure 1A**).

We further compared the sequence polymorphism levels among effector genes of RxLR, CRN, NLP, and elicitin families, as well as house-keeping genes (160, 25, 42, 32, and 320 genes were analyzed, respectively; Supplementary Table S4). The different groups of effector genes likely had lower sequence identities than the cases of all genes, while the group of house-keeping genes was more conserved with identity values significantly greater than those of all genes (**Figure 1A**). In addition, the average identities of the aligned sequences from non-gene regions were all lower than those of whole genome and gene regions; they were 98.981% for P7074 vs. P6497, 99.083% for P7064 vs. P6497, and 99.181% for P7076 vs. P6497 (**Figure 1A**). However, in summary, all these results were consistent for their sequence similarity relationship.

To further determine the relationship among the four isolates, we aligned the CDSs of the 2,626 genes, sequences of which could be identified in all four isolates, and concatenated the sequences one-by-one into a super-sequence for each isolate. As the constructed Maximum-Likelihood (ML) phylogenetic tree shows (**Figure 1C**), the relationship among the four isolates was also consistent with results from all abovementioned sequence comparisons.

### Candidate Genes under PS or NS

To determine the number of polymorphic genes under evolutionary selection, rates of non-synonymous (dN) and synonymous (dS) nucleotide substitutions were calculated and compared for every allele pair using the YN00 program integrated into the PAML4.7 software package (Yang, 2007). We found that 68 pairs showed dN/dS > 1.2 and *P* < 0.1 (Student’s *t*-test), and 55 pairs showed dS/dN > 1.2 and *P* < 0.1 between P7064 and P6497. Due to a relatively relaxed cutoff of *P*-values, these genes were suggested as preliminary candidate PS genes and NS genes, respectively. In addition, the numbers of gene pairs were 51 and 35 for P7074 vs. P6497, respectively, and 52 and 65 for P7076 vs. P6497, respectively. Based on the combined results, we obtained 153 PS genes and 139 NS genes (**Figures 1D,E**, Supplementary Tables S3 and S5). There were the least proportions of PS and NS genes (5.0 and 6.2‰, respectively) identified from the analysis of P7076-P6497 allele pairs, and greater and similar proportions from the analysis of P7064-P6497 (PS: 9.8‰ > 5.0‰, χ^2^ test *P-*value < 0.001; NS: 7.9‰) and P7074-P6497 (PS: 10.3‰ > 5.0‰, *P* < 0.01; NS: 7.1‰) allele pairs (**Figures 1D,E**). Again, these results were consistent for their sequence similarity relationship (**Figures 1A,C**).

**FIGURE 2:**
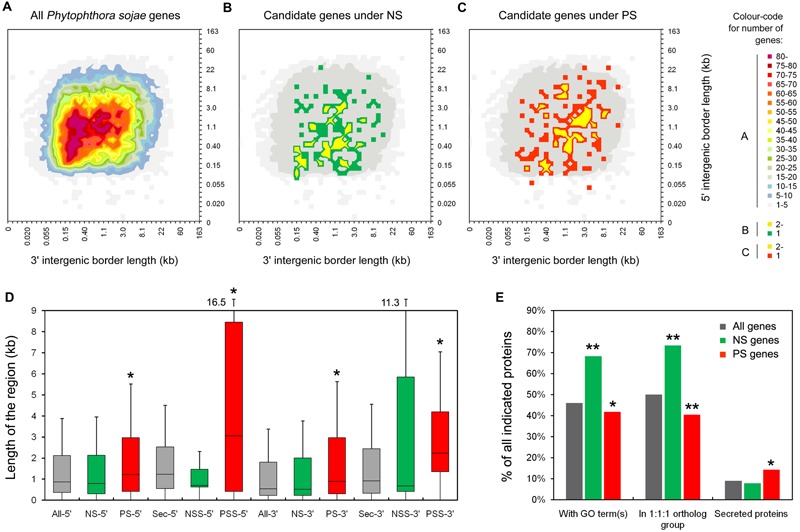
**Characteristics of the candidate PS and NS genes. (A–C)** Distribution of intergenic region lengths of all *P. sojae* genes **(A)**, NS genes **(B)**, and PS genes **(C)**. **(D)** Boxplots showing the length of the 5′ and 3′ intergenic regions for all genes (All-), NS genes (NS-), PS genes (PS-), all secreted protein encoding genes (Sec-), secreted protein encoding NS genes (NSS-), and secreted protein encoding PS genes (PSS-) in *P. sojae*. Values inside each box correspond to the middle 50% of the data (between the 25th [Q1] and 75th [Q3] percentiles) and the line within the box represents the median. The whisker ends reach Q3+1.5IQR and Q1-1.5IQR (IQR = Q3-Q1), respectively. A significant difference between two sets of data (with a *P* < 0.05 from a Wilcoxon signed ranks test, using data of all genes as a reference), were marked with an asterisk. **(E)** Proportions of genes annotated with GO terms, in the 1:1:1 ortholog group, and predicted to encode classic secreted proteins. Comparing to “all genes”, data with significant differences (χ^2^ test) are marked with a single asterisk (*P* < 0.05) or double asterisks (*P* < 0.01).

To elucidate the gene density of genome regions where certain PS and NS genes localize, intergenic region lengths (IRLs) were plotted (**Figures 2A–C**) and compared. We found median 5′ and 3′ IRLs for NS genes that were both smaller (but not significant) than those of all *P. sojae* genes (5′ IRL: 1.15 kb < 1.17 kb; 3′ IRL: 0.65 kb < 0.80 kb; **Figure 2D**). In contrast, both values for PS genes were significantly greater than all of the *P. sojae* genes (5′: 1.47 kb > 1.17 kb; 3′: 1.23 kb > 0.80 kb; **Figure 2D**). Furthermore, the differences in IRLs among the three sets of genes were enlarged when only considering the predicted secreted protein-encoding genes (**Figure 2D**). The results indicated that the PS genes, particularly the secreted protein-encoding genes, were more concentrated at the ‘plastic regions’ of the genome (Haas et al., 2009; Raffaele and Kamoun, 2012).

In addition, 68% (95/139) of the NS genes could be mapped t gene ontology (GO) terms, which was significantly greater than that of all *P. sojae* genes (8,741/19,027 = 46%). In contrast, the proportion for PS genes was smaller than that for all *P. sojae* genes (64/153 = 42%; **Figure 2E**). We also compared the proportions of genes showing 1:1:1 orthology relationships among *P. sojae, P. ramorum*, and *P. infestans*, designated ‘core proteome’ (Haas et al., 2009). Consistent with the results of GO analysis, higher proportion of the NS genes (102/139 = 73%) belonged to the core proteome, which was greater than the proportion for all of the *P. sojae* genes (9,510/19,027 = 50%), and even greater than the PS genes (62/153 = 41%; **Figure 2E**). The abovementioned results indicated that the genes, which have more general functions and are more conserved among *Phytophthora* species, were probably more affected by NS, whereas the species-specific and/or less conserved genes were more likely to have been affected by PS.

Among the GO-mapped genes, the proportions of genes mapping to the different GO terms were compared. Generally, the NS and PS genes targeted a wide range of different categories of GO terms. However, the NS genes were overrepresented in catalytic function and metabolic process, in contrast to the PS genes. In addition, the PS genes were overrepresented in the molecular function of binding (**Supplementary Figure S3**).

### Transcription Patterns of the Identified Genes

**FIGURE 3:**
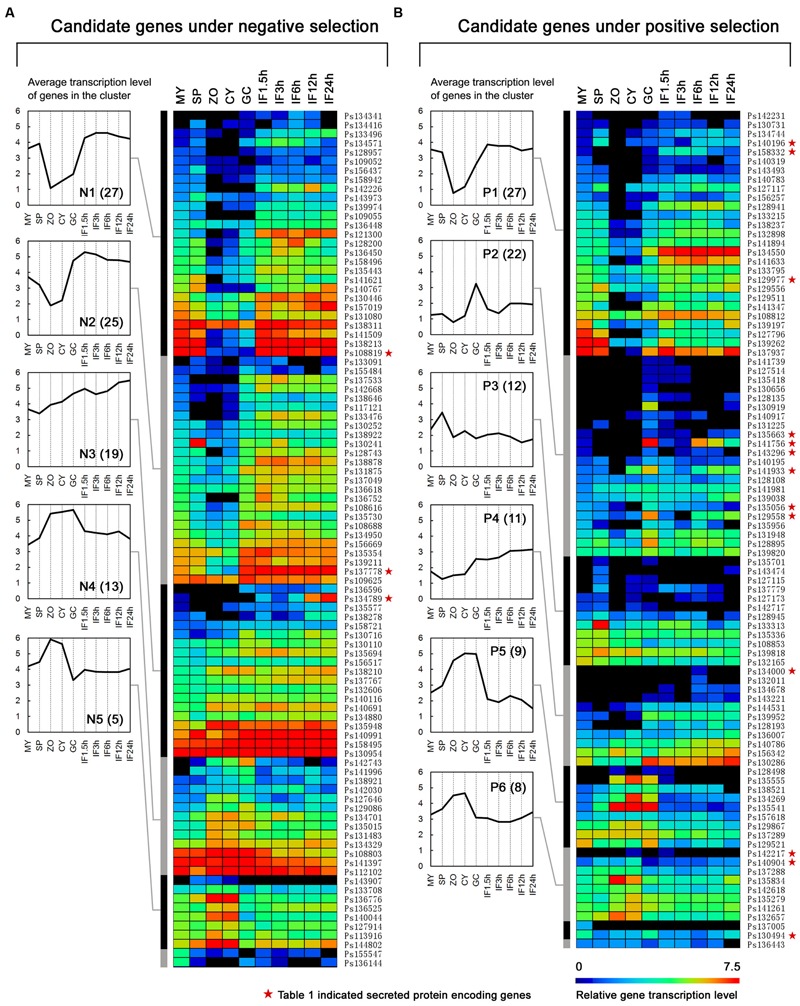
**Transcription patterns of the candidate NS genes **(A)** and PS genes **(B)**.** The heat map shows gene transcription patterns in 10 stages of the *P. sojae* life cycle: mycelia (MY), zoosporangia (SP), zoospores (ZO), cysts (CY), germinating cysts (GC), and samples from 1.5, 3, 6, 12, and 24 h post-inoculation of soybean leaves (IF1.5h to IF24h). The color bar represents log_2_ values of gene transcripts per million reads (TPM), ranging from dark blue (0) to red (7.5). Black represents no detected transcript. Transcription patterns are grouped into five (N1–N5) and six (P1–P6) major clusters using HCL methods and MEV software. The average gene transcription levels of these clusters are plotted and shown on the left of the heatmap. Gene numbers in each cluster are displayed in parentheses. Red stars mean the genes are also described in **Table 1**.

The transcription patterns of the genes were analyzed and compared on the basis of the available digital gene expression (DGE) profiling data for 10 stages during *P. sojae* development and infection (Ye et al., 2011). We found more NS genes (NS genes, 94/139 = 68%; PS genes, 92/153 = 60%) that could be detected in at least one stage. Transcription levels of the detected NS genes were obviously higher than those for the PS genes (**Figures 3A,B**). Thus the PS genes were transcribed at a relatively lower level and possibly not transcribed in the sampled stages.

Based on the hierarchical clustering (HCL) method, we obtained five (N1–N5; **Figure 3A**) and six (P1–P6; **Figure 3B**) major transcription patterns for the detected NS genes and PS genes, respectively. Three groups representing 48% NS genes and 48% PS genes displayed similar transcription patterns, such as N1-P1, N4-P5, and N5-P6; dramatic changes in transcription levels of these genes were mostly associated with the stages of zoospores and cysts (**Figures 3A,B**). There were two NS- and three PS specific clusters, respectively. The N2 and N3 clusters contained 25 and 19 genes, respectively, which exhibited an increased number of transcripts during infection; genes in N2 cluster also exhibited decreasing transcripts during stages of zoospores and cysts (**Figure 3A**). The P2, P4, and P3 clusters contained 22, 11, and 12 PS genes which exhibited abundant transcripts during certain stages, namely cyst germination, cyst germination and soybean infection, and zoosporangia, respectively (**Figure 3B**). Interestingly, the transcription pattern of P2 cluster genes (24% of detected PS genes) was similar to that of RxLR effector genes in a previous report based on the same dataset (**Figure 3B**; Ye et al., 2011).

### Characteristics of the Secreted Protein Encoding Genes

Effectors are secreted proteins of pathogens that could alter the host to advance the infection process (Wawra et al., 2012). We found 14% (22/153) of PS genes were predicted to encode secreted proteins, which was significantly greater than that for all *P. sojae* proteins (9%, 1,762/19,027) and NS genes (8%, 11/139; **Figure 2E**). The PS and NS genes predicted to encode secreted proteins were termed PSS and NSS genes, respectively. Among these genes, 15 PSS genes and 5 NSS genes, having no non-self identical sequences among all reference genes (based on an all vs. all BLASTN, with an *e*-value of 1e-40 as a cut-off), were suggested to have the least possibility of false mapping with paralogous genes. Thus they were further described.

Among the five NSS genes, three genes were transcriptionally detected (**Table 1**). Except for *Ps137778* (an incorrect gene model), *Ps108819* encoded an ortholog of the cellulose-binding elicitor lectin (CBEL) in *P. parasitica* (Mateos et al., 1997), which was revealed to be a novel pathogen-associated molecular pattern (PAMP) in oomycetes that are targeted by the innate immune system of plants (Gaulin et al., 2006). This finding likely supported the hypothesis that many elicitors should be encoded by core genes that are under strong negative selection (McCann et al., 2012). In addition, *Ps134789* was predicted to encode a pectinacetylesterase; however, the activity and function of this protein family in plant pathogens are unknown. Conserved sequences of both genes could be clearly identified from genome assembly of *P. melonis*, a close species of *P. sojae*. Interestingly, significant indication of PS (dN > dS) was observed for both orthologous gene pairs (data not shown).

**Table 1:** Information of the candidate genes which encode secreted proteins.

Gene ID	Polymorphism in P7064^a^	Polymorphism in P7074^a^	Polymorphism in P7076^a^	Predicted protein functions	Gene expression cluster^bc^
***Genes under negative selection* (5)**					
Ps108819	Unknown	Unknown	0.32 (0.086)	Ortholog of *P. parasitica* CBEL	N1
Ps137778	Unknown	0.64 (0.007)	Unknown	Eukaryotic translation initiation factor	N2
Ps127025	Unknown	0.36 (0.047)	0.36 (0.047)	Isoamyl acetate-hydrolyzing esterase	–
Ps134789	No	0.53 (0.083)	No	Pectinacetylesterase	N3
Ps140419	No	0.56 (0.080)	No	Unknown	–
***Genes under positive selection* (15)**					
Ps140196	2.46 (0.000)^∗^	Unknown	No	RxLR family protein, Avh158	P1
Ps129558	3.27 (0.000)^∗^	Unknown	No	RxLR family protein, Avh181	P2
Ps134000	3.20 (0.001)^∗^	Polymorphic	No	RxLR family protein, Avh229	P4
Ps143296	1.81 (0.048)	No	No	RxLR family protein, Avh292	P2
Ps141933	3.69 (0.000)^∗^	2.38 (0.001)^∗^	No	RxLR family protein, Avh163	P2
Ps140904	Unknown	Unknown	1.30 (0.046)	RxLR family protein, Avh165	P6
Ps141756	Polymorphic	Unknown	3.80 (0.005)^∗^	RxLR family protein, Avh238	P2
Ps143551	Polymorphic	Unknown	1.54 (0.082)	Necrosis-inducing-like protein	–
Ps130494	Unknown	Polymorphic	1.21 (0.080)	Glycoside hydrolase	P7
Ps135056	1.30 (0.078)	Unknown	Unknown	Histidine acid phosphatase	P2
Ps142217	1.89 (0.009)	Polymorphic	No	Neutral zinc metallopeptidase	P6
Ps158332	No	1.60 (0.046)	No	TPR repeat-containing protein	P1
Ps135663	Polymorphic	1.48 (0.086)	No	Hypothetical with ANK domain	P2
Ps133322	Unknown	Unknown	1.64 (0.084)	Hypothetical with ANK domain	–
Ps129977	Polymorphic	1.32 (0.075)	Polymorphic	Hypothetical with ARM domain	P1

*^a^The data are polymorphism compared to the reference isolate P6497. Digits on the left are the values of dN/dS; digits in the brackets are the *P*-values from Student’s *t*-test; asterisk indicates a stronger PS, i.e., *P-*value < 0.05 (after Bonferroni correction) and dN/dS > 2. ^b^Consistent with those displayed in **Figure 3**. “–”means expression of the gene was not detected or unknown, thus not shown in **Figure 3**. ^c^More information of these genes are in Supplementary Tables S3 and S5.*

Among the 15 PSS genes, 8 genes belonged to the well-known effector families, including seven RxLRs and one NLP (i.e., *PsAvh158*, -*163*, -*165*, -*181*, -*229*, -*238*, and -*292*, and *PsNLP7*) (**Table 1**). Except for *Ps140904* (an incorrect gene model for *PsAvh165*), these PS genes had previously been identified for PS in special studies of these effector families (Wang et al., 2011; Dong et al., 2012), suggesting that the global analysis method used in this study was useful. The transcription pattern cluster P2 contained the most PSS genes; there were six genes (38%), including four RxLR effector genes (*PsAvh163*, -*181*, -*238*, and -*292*) (**Table 1**). Genes in this cluster were observed to exhibit increased transcripts during the cyst germination and infection stages (**Figure 3B**). Five PSS genes had stronger indication of PS, i.e., dN/dS > 2 and *P* < 0.05 (after Bonferroni correction), and all of the five genes were RxLR effector genes (*PsAvh158*, -*163*, -*181*, -*229*, and -*238*). These results suggest that the RxLR effector genes might be dominant targets that are affected by strong PS stress in the *P. sojae* genome. Intense host–pathogen interaction might be one of the major sources of stress in pathogen evolution.

### Candidate Genes with Presence/Absence Polymorphism

In the above results, we found there were between 99.5 and 99.8% reference genes in P6497 having matched sequences in assemblies of the other *P. sojae* isolates. Therefore, the remaining 60, 93, and 42 genes were suggested to be candidate genes that might be absent in the P7064, P7074, and P7076 genomes, respectively. Based on the combined results, we obtained 134 candidate genes; there were 24, 13, and 97 genes be absent in the three genomes, two genomes, and one genome, respectively (Supplementary Table S6). These genes were not overlapped with any of the PS or NS genes.

Two RxLR genes (*PsAvr1d* and *PsAvh245*) were identified that had presence/absence polymorphism. The absence of *PsAvr1d* in P7074 and P7076 genomes has been revealed (Wang et al., 2011; Na et al., 2013; Yin et al., 2013). *PsAvh245* exhibited an absence in genomes of all three non-P6497 isolates; however, no transcript was detected for this gene in all sampled stages (Ye et al., 2011).

Among the 134 genes (Supplementary Table S6), their median 5′- and 3′ IRLs were greater (but not significant) than those of all *P. sojae* genes (5′: 1.75 kb > 1.15 kb, Wilcoxon signed ranks test *P-*value = 0.09; 3′: 0.89 kb > 0.65 kb, *P* = 0.13). Twelve genes were predicted to encode secreted proteins; the proportion (9%) was equal to that of all *P. sojae* genes. However, only 39 genes (29%) were detected in the transcriptome data (Ye et al., 2011), 28 genes (21%) had annotated GO terms, and 20 genes (15%) belonged to the ‘core proteome’; these proportions were all even lower than the abovementioned lowest case in PS genes. These results indicated that these candidate *P. sojae* genes of presence/absence polymorphism were much less conserved, more likely to be species-specific, and many of them might be non-transcribed “dead” genes. In other words, gene lost and gene death (or silencing) might be alternative; they are the ways of natural selection other than PS and NS during *P. sojae* genome evolution.

## Discussion

We found that the genome sequences among four representative *P. sojae* isolates were over 99.6% in identity. No more than 10% of the identified allele pairs had over three mismatches or gaps. Only approximately 1–2% of the *P. sojae* genes was predicted to be affected by evolutionary selection. In contrast to the NS genes, more PS genes located within the genome regions that had relatively larger intergenic distance. Half of the identified PSS genes were among the known effectors. These results might support a conclusion that, among different isolates of *P. sojae*, core genome sequences are generally conserved, whereas sequences in the ‘plastic regions’ are relatively more variable. The PS or effector genes were more likely to be located at the plastic regions and to be major objects of PS. Although feature of expanded genome with larger intergenic distances and the ‘plastic regions’ of genome are not obvious in *P. sojae* than in *P. infestans*, with a model of ‘two-speed genome’ in filamentous pathogens (Haas et al., 2009; Dong et al., 2015), the genome of *P. sojae* is likely under a similar evolution pattern.

We identified a similar number of candidate genes affected by PS (153) and NS (139), however, the secreted protein-encoding genes were more frequently present in PS than NS genes, at a rate of twofold to threefold. Furthermore, RxLR effector genes took almost half of the PSS genes and exhibited much stronger levels of PS. As the largest effector family currently known in *Phytophthora*, RxLR effector family might be one of the dominant targets that are affected by PS stress among the *Phytophthora* effector genes. In addition, many PS and PSS genes were transcriptionally infection-related. These results may support that the intense host–pathogen interaction is one of the major stresses in genome evolution of pathogens.

Based on our available genomic data and analysis method, we identified some genes under PS or with presence/absence polymorphism that were previously reported (Wang et al., 2011; Dong et al., 2012; Na et al., 2013; Yin et al., 2013), indicating a good efficiency of the method. However, this study still could not avoid some limitations. The assembly sizes of P7064, P7074, and P7076 (51–64 Mb) were quite far from the 95 Mb of the reference isolate P6497. The majority of missing sequences were likely from non-coding and repetitive regions. This may cause a bias in the sequence variation analysis of genome, especially that of the non-coding regions. Due to deficient sequencing depth and limited length of the reads, the assembled contigs for P7064, P7074, and P7076 were generally too short; in addition, 40% *P. sojae* genes had non-self similar sequence in the genome. These problems might restrict the accuracy of sequence mapping. We had to use strict filtering criteria and discarded a lot of contigs with ambiguous mapping and/or poor alignment coverage; finally the reserved alignments only covered 26 to 56% of all *P. sojae* genes. This means that some of the potentially most interesting variation might not been investigated. For example, contigs with multiple best matches could represent regions with high duplication rates, which is frequent in effector-bearing regions. In addition, contigs with incomplete alignment coverage could include genes with really high polymorphism preventing alignment, or with insertions and/or deletions.

The abovementioned problems on identification of allele sequences could somewhat be solved through manual methods. For example, based on the same data, we had identified allele sequences for 97% of all *P. sojae* RxLR effector genes; and there were 18 PS genes (Wang et al., 2011). However, the case that gene and its intergenic regions have multiple highly similar sequences in genome might still have the problem. In addition, the polymorphism was analyzed according to the assembled genome sequences, which are not the natural sequence. Some of the assemblies might be heterozygous consensus sequences and some might also have errors related to 454 sequencing technology. Therefore, the further studies of *P. sojae* genome polymorphism require additional higher-quality genome data.

However, in summary, this study has provided a landscape of genome variation and evolutionary pattern among the four representative isolates of *P. sojae*. The results suggest a relatively covert two-speed genome evolution pattern in *P. sojae* and will provide clues for identification of new virulence factors in the oomycete plant pathogens.

## Author Contributions

YcW, BT, and WY conceived the study, BT provided the sequence data, YcW and BT provided the bioinformatic servers, WY and YW performed the bioinformatic analysis, and WY wrote the paper.

## Conflict of Interest Statement

The authors declare that the research was conducted in the absence of any commercial or financial relationships that could be construed as a potential conflict of interest.

## Footnotes

1 http://genome.jgi.doe.gov

2 http://phy.njau.edu.cn/ptd

## Supplementary Material

FIGURE S1 **Flow chart of the genomic analysis**.

FIGURE S2 **Proportion of genes with total mismatch and gap sites between allele pairs**.

FIGURE S3 **Proportions and numbers of genes corresponding to different GO categories.** Compared to that of all genes; data with significant differences (χ^2^ test: *P < 0.01*) are marked with an asterisk.
