# Supplementary figures and images for: Comparative Genomic Analysis among Four Representative Isolates of Phytophthora sojae Reveals Genes under Evolutionary Selection
Source: Front Microbiol. 2016 Sep 30;7:1547. doi: 10.3389/fmicb.2016.01547 (PMC5042962; doi:10.3389/fmicb.2016.01547)

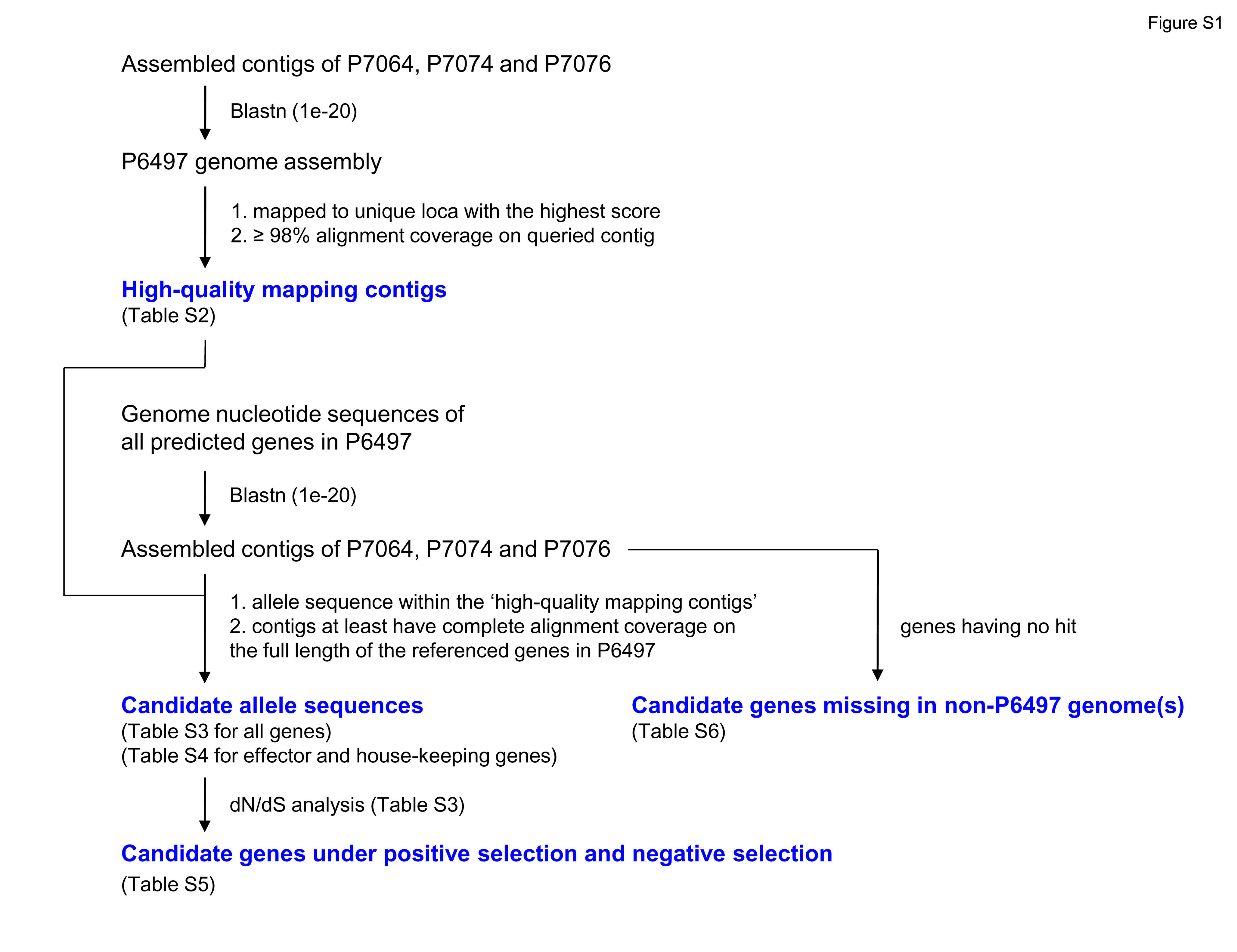

Supplement: FIGURE S1 — Flow chart of the genomic analysis. [file Image_1.JPG]

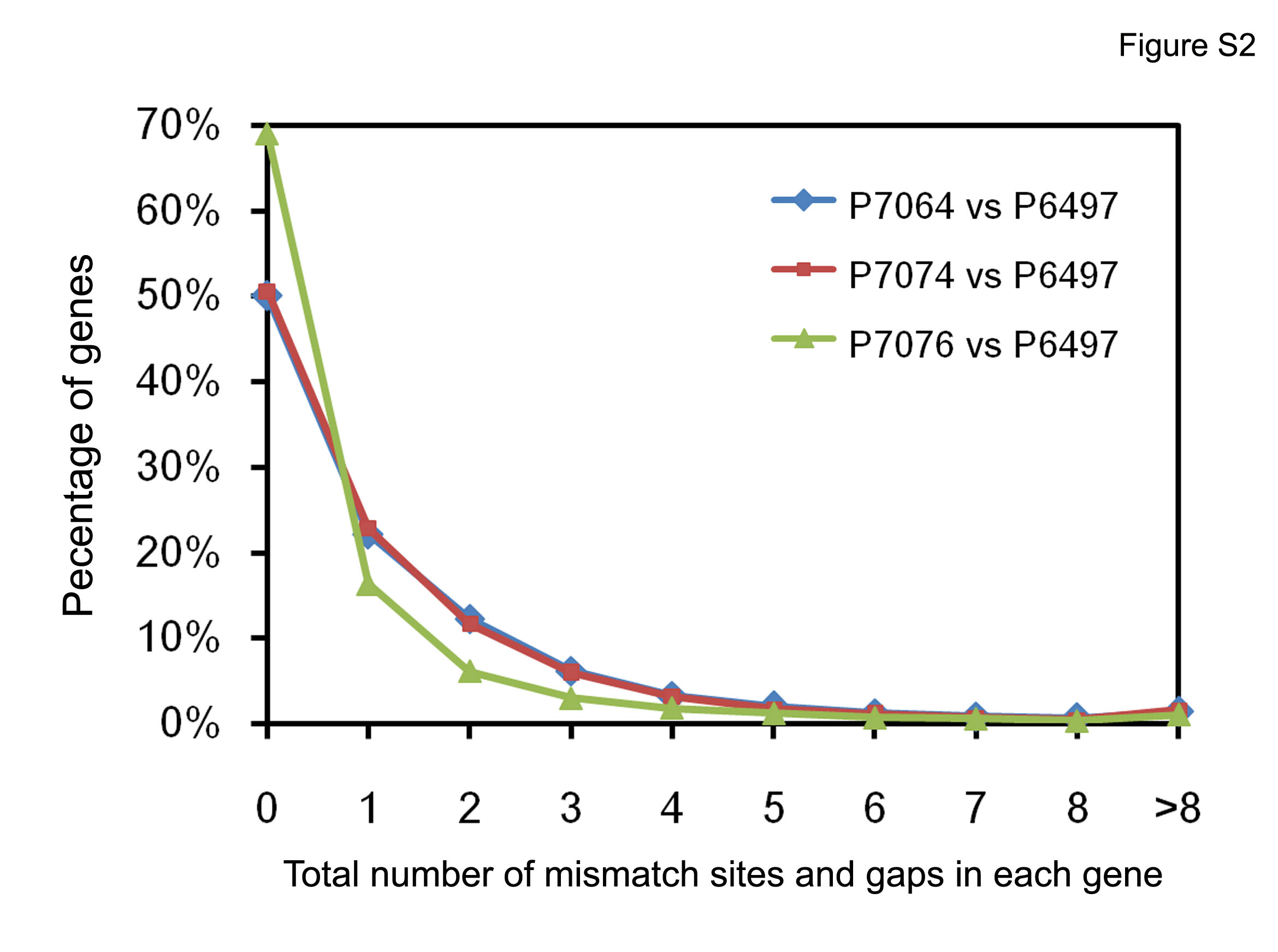

Supplement: FIGURE S2 — Proportion of genes with total mismatch and gap sites between allele pairs. [file Image_2.JPEG]

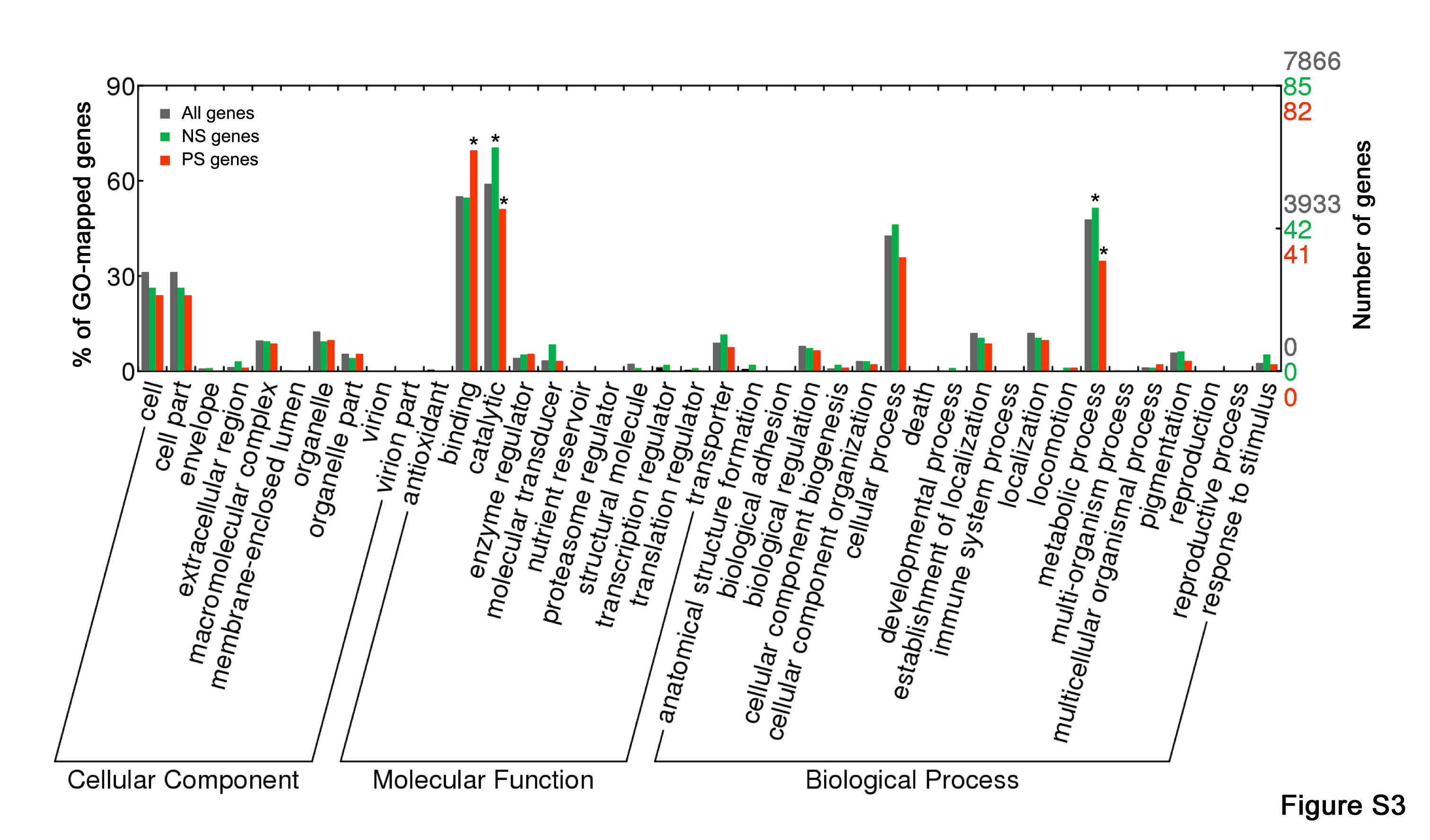

Supplement: FIGURE S3 — Proportions and numbers of genes corresponding to different GO categories. Compared to that of all genes; data with significant differences (χ2 test: P < 0.01) are marked with an asterisk. [file Image_3.JPEG]
